# Cerebral dopamine neurotrophic factor for spinal cord injury: Targeting JNK1 to relieve neuroinflammation and improve neural repair

**DOI:** 10.4103/NRR.NRR-D-24-00890

**Published:** 2025-06-19

**Authors:** Yanxiao Xiang, Pengchao Du, Yayun Zhang, Hao Li, Songgang Wang, Xianlei Gao, Xin Pan, Hua Zhao

**Affiliations:** 1 Department of Pharmacy, Qilu Hospital of Shandong University, Cheeloo College of Medicine, Shandong University, Jinan, Shandong Province, China; 2 School of Basic Medical Sciences, Binzhou Medical University, Yantai, Shandong Province, China; 3 Department of Orthopedic Surgery, Tongji Hospital, Tongji Medical College, Huazhong University of Science and Technology, Wuhan, Hubei Province, China; 4 Department of Orthopedics, Qilu Hospital of Shandong University, Cheeloo College of Medicine, Shandong University, Jinan, Shandong Province, China

**Keywords:** cerebral dopamine neurotrophic factor, ER stress, JNK1, microenvironment, microglia, nerve repair, neural regeneration, neuroinflammation, proinflammatory cytokine, spinal cord injury

## Abstract

Previous studies have shown that endoplasmic reticulum stress induces neuronal apoptosis, necrosis, and pro-inflammatory microenvironment after spinal cord injury. The JNK pathway is activated by endoplasmic reticulum stress and reactive oxygen species. Our previous research demonstrated that cerebral dopamine neurotrophic factor has anti-inflammatory effects and promotes the repair of the damaged spinal cord after injury. However, the molecular mechanism remains unclear. In this study, we found that cerebral dopamine neurotrophic factor binds JNK1 and regulates JNK1/2-c-Jun-p53 signaling in lipopolysaccharide-induced microglia. Cerebral dopamine neurotrophic factor also alleviated neuroinflammation by reducing the secretion of pro-inflammatory cytokines. Overexpression of cerebral dopamine neurotrophic factor in a mouse model of spinal cord injury promoted nerve regeneration and motor function recovery. These findings indicate the possibility for cerebral dopamine neurotrophic factor treating spinal cord injury by targeting the JNK1/2-c-Jun-p53 pathway.

## Graphical abstract

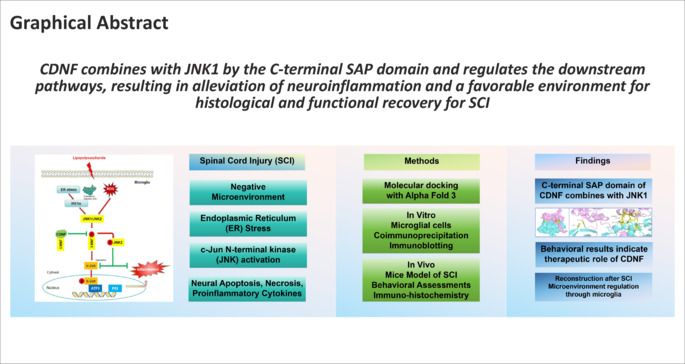

## Introduction

Spinal cord injury (SCI) leads to the death of neurons and damage to axons, resulting in decreased motor and sensory abilities. Nerve recovery and regeneration after SCI are extremely difficult to achieve because of the adverse environment caused by injury-related processes such as neuroinflammation, lipid peroxidation, ischemia, and glial scarring (Nashmi and Fehlings, 2001; Parr et al., 2008; Wright et al., 2011). During neuron death, protein misfolding and aggregation lead to endoplasmic reticulum (ER) stress and mitochondrial dysfunction, resulting in apoptosis of neurons and immuno-inflammatory responses. Neuron death and the secondary inflammatory microenvironment are believed to hinder recovery and repair because of the development of additional injuries and the extension of harm to areas near the damaged spinal cord through inflammatory cytokines (Hellenbrand et al., 2021). Numerous studies have concentrated on comprehending the microenvironment and subsequent tissue damage following SCI to identify potential therapeutic targets that can effectively promote neural recovery (Fan et al., 2018; Shu et al., 2020; Liu et al., 2023).

Following SCI, the homeostasis of the ER in glial cells is disrupted and the unfolded/misfolded protein responses are induced, as known as ER stress. Studies have demonstrated the role of ER stress in ischemia–reperfusion (I/R) injury, highlighting the involvement of apoptosis, inflammation, and autophagy (Fanger et al., 1997; Widmann et al., 1999; Cargnello and Roux, 2011). ER stress also leads to the activation of glial cells such as microglia and astrocytes and c-Jun N-terminal kinase (JNK) pathways (Xue et al., 2022). Activated glial cells secrete proinflammatory cytokines such as prostaglandin E2 (PGE2), tumor necrosis factor alpha (TNF-α), and interleukin-1 beta (IL-1β), which further deteriorates the microenvironment (Cheng et al., 2013).

In the pathological processes of injury, excessive reactive oxygen species and nitric oxide cause phosphorylation and nucleus translocation of JNK, which phosphorylates the transcription factor Jun (c-Jun), leading to the expression of multiple inflammation and apoptosis-related genes (Shvedova et al., 2018). A previous study showed that ER stress after I/R injury activates JNK through IRE1 (Xue et al., 2022). Activation of the IRE1/JNK pathways in tubular epithelial cells leads to secretion of inflammatory cytokines. Suppression of IRE1/JNK signaling was protective against myocardial cell inflammation induced by low-dose lipopolysaccharide (LPS) (Wu et al., 2019; Liang et al., 2020).

Targeting inflammation is a promising therapeutic method for the treatment of SCI and nerve regeneration (Saad et al., 2025; Zhang et al., 2025). Large doses of glucocorticoids are commonly administered after traumatic SCI; however, the use of glucocorticoids is controversial because of their side effects (Miller, 2008; Markandaya et al., 2012; Bydon et al., 2014). Cerebral dopamine neurotrophic factor (CDNF), as a protective factor, has demonstrated restorative properties in various models of neuropathology, for instance, Parkinson’s disease and other degenerative neural diseases (Pakarinen and Lindholm, 2023). CDNF is localized mainly in the cavity of the ER, and its primary function is to regulate ER stress, enhancing cell survival by lowering ER stress, inflammation, apoptosis, and aggregation of toxic oligomers (Lõhelaid et al., 2024). Our previous research revealed that CDNF decreased the secretion of inflammatory cytokines such as PGE2 and IL-1β during microglial inflammation induced by LPS exposure (Cheng et al., 2013). The protective effect of CDNF against inflammation partly results from the suppression of JNK pathways, which were related to ER stress. The specific mechanism by which CDNF regulates neuroinflammation and promotes neural restoration is still not understood.

This study investigated the potential of CDNF in regulation of microglial inflammation induced by LPS and the functional recovery *in vivo* followed by spinal cord injury. We also examined the interaction between CDNF and JNK1 and the effects of CDNF on downstream JNK1 signaling.

## Methods

### Primary microglial culture

The animal experimental protocol was approved the Ethics Committee of Qilu Hospital of Shandong University (approval No. KYLL-2022(ZM)-519) and animal experiments were performed in accordance with the Guide for the Care and Use of Laboratory Animals (National Research Council, 2011).

BALB/c mouse pups (1 day post-natal, *n* = 10) were provided by the Experimental Animal Center of Shandong University (SYXK (Lu) 2023003) in China. After anesthesia by inhalation of 3% isoflurane for induction and 1.5% isoflurane for maintenance (Lunan Pharmaceuticals Co., Ltd., Linyi, China), cortical tissues were mechanically separated from the meninges and white matter, cut into fragments, and seeded in flasks (25 cm^2^, 1 × 10^6^/mL). Following a 3-day incubation, the samples were agitated at 250 × *g* overnight to isolate the microglia and astrocytes. Microglia that were floating were collected from the supernatants and placed in flasks for cultivation. After the cells had fully attached, the medium was changed to eliminate non-adherent cells and acquire extremely pure primary microglia, as described in previous research (Zhang et al., 2019). Cells passaged 2–3 times were used in experiments.

### Treatment of primary microglia

Primary microglia were divided into four treatment groups: Control, LPS, LPS + CDNF, and LPS + CDNF-shRNA. Microglia (5 × 10^6^/mL) were plated in 6-well plates (Corning, New York, NY, USA) with 10% fetal bovine serum (Gibco, Thermo Fisher Scientific Inc., Cambridge, MA, USA). After 24 hours, the solution was changed to 1 mL of medium without fetal bovine serum, and the cells were grown for another day. The LPS group was stimulated with 1 µg/mL LPS (Beyotime Biotechnology, Shanghai, China) for 24 hours before harvest. The LPS + CDNF-shRNA group was transduced by lentivirus expressing CDNF-shRNA (Genechem, Shanghai, China) at a rate of five multiplicity of infection for 72 hours. The cell medium was changed, and LPS was added at 1 µg/mL for 24 hours. The LPS + CDNF group was pretreated with CDNF (200 ng/mL; Peprotech, Thermo Fisher Scientific Inc.) for 1 hour and then stimulated with LPS for 24 hours. The concentrations of reagents were determined in previous research (Zhao et al., 2014). The cell and animal experiments are shown in **Additional Figure 1**.

**Figure 1:**
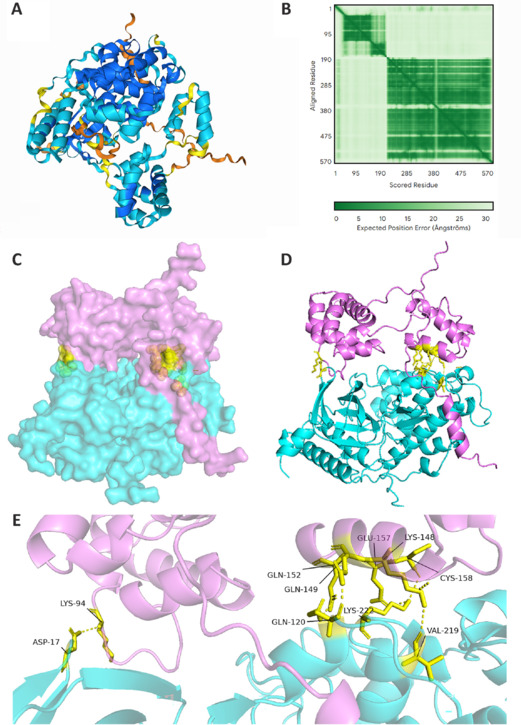
Molecular docking analysis of CDNF and JNK1. (A) The spatial structures of CDNF and JNK1 were generated by AlphaFold3 (blue, very high, pLDDT > 90; turquoise, confident, 90 > pLDDT > 70; yellow, low, 70 > pLDDT > 50; orange, very low, pLDDT < 50). (B) Expected position error assessment. Lower scores shown as dark green indicate higher confidence with less error; the higher scores shown as bright green indicate lower confidence with more predicted error. (C) The interactions between CDNF (violet) and JNK1 (turquoise) are highlighted with yellow in the surface image of three-dimensional spatial structures. (D) The hydrogen bonds connecting the two molecules are highlighted in yellow. (E) Five residues from the C-terminal SAP domain of CDNF (LYS-148, GLN-149, GLN-152, GLU-157, CYS-158) interact with three residues of JNK1 (GLN-120, VAL-219, LYS-222). Only one residue, LYS-94, from the N-terminal saposin-like domain of CDNF, interacts with JNK1 at ASP-17. ASP: Aspartate; CDNF: cerebral dopamine neurotrophic factor; CYS: cysteine; GLN: glutamine; GLU: glutamine; JNK: c-Jun N-terminal kinase; LYS: lysine; pLDDT: predicted local distance difference test; VAL: valine.

### Molecular docking analysis

The docking calculations of CDNF and JNK1 were performed using AlphaFold3 (https://alphafoldserver.com/; Abramson et al., 2024) following the instructions (Roy and Al-Hashimi, 2024). The molecular interaction between mouse JNK1 (JIP1, 707 amino acids) and mouse CDNF (Armetl1, 187 amino acids) was examined with AlphaFold3. The results were displayed with PyMOL2.5.7 (Schrödinger, Inc., NY, USA). The spatial conformation of the two molecules was displayed and the accuracy was scaled by predicted local distance difference test (pLDDT), a per-atom confidence estimate on a 0–100 scale where a higher value indicates higher confidence. pLDDT aims to predict a modified LDDT score that only considers distances to polymers. Different colors stand for four scales of confidence (blue, very high, pLDDT > 90; turquoise, confident, 90 > pLDDT > 70; yellow, low, 70 > pLDDT > 50; orange, very low, pLDDT < 50). The predicted aligned error (PAE) estimates the errors in the relative position and orientation between two tokens in the predicted structure. Higher scores of expected position error (bright green) indicate higher predicted error and lower confidence, whereas lower scores (dark green) indicate higher confidence.

### Western blot analysis

Microglia were washed twice with ice-cold phosphate-buffered saline and lysed in lysis buffer. The cell debris was removed by centrifugation (5 minutes, 4°C, 12,000 × *g*). Equal quantities of protein (10 µg) were separated by 10% sodium dodecyl-sulfate polyacrylamide gel electrophoresis and transferred to polyvinylidene fluoride membranes.

The membranes were incubated with antibodies against JNK1, JNK2 and GAPDH for 12 hours followed by incubation with secondary antibodies conjugated with horseradish peroxidase for 1 hour at 20°C. All antibodies are listed in **Additional Table 1**. The bands of immunoblots were observed with a Western Blot Imaging Machine (Thermo Fisher Scientific Inc.).

**Additional Table 1:** The antibodies used in this study

Antibody	Species	Dilution	Supplier	Cat#	RRID
CDNF	Goat	WB: 1:500 IF: 1:100	Thermo Fisher, Cambridge, MA, USA	PA5-47708	AB_2606900
JNK1	Mouse	WB: 1:500	R&D Systems, Minneapolis, MN, USA	MAB17761	AB_2235014
JNK2	Mouse	WB: 1:500	R&D Systems	MAB1846	AB_2141157
Phosphor-JNK	Rabbit	WB: 1:500 IF: 1:100	Cell Signaling, Danvers, MA, USA	4668	AB_823558
SAPK/JNK	Rabbit	WB: 1:500	Cell Signaling	9252	AB_2250373
GAPDH	Rabbit	WB: 1:500	Abcam, Cambridge, MA, USA	ab9485	AB_307275
NF	Rabbit	IHC: 1:200	Beijing Biosynthesis Biotechnology Co., Ltd., Beijing, China	bs-0708R	AB_2565456
NeuN	Rabbit	IF: 1:100	Cell Signaling	24307	AB_2651140
Anti-rabbit IgG	Goat	WB: 1:500	Cell Signaling	7074	AB_2099233
Anti-rabbit IgG	Goat	IHC: 1:1000	Beijing Biosynthesis Biotechnology Co., Ltd.	bs-40295G-IRDye8	AB_3076685
Anti-goat IgG-FITC	Mouse	IF:1:500	Santa Cruz, Dallas, TX, USA	sc-2356	AB_628489
Anti-rabbit IgG-Alexa 555	Goat	IF: 1:500	Thermo Fisher	A-21434	AB_141733
Iba-1-Alexa Fluor 594	Mouse	IF: 1:100	Santa Cruz	sc-32725	AB_667733

CDNF: Cerebral dopamine neurotrophic factor; FITC: fluorescein isothiocyanate; GAPDH: glyceraldehyde-3-phosphate dehydrogenase; IF: immunofluorescence staining; IHC: immunohistochemistry; JNK: c-Jun N-terminal kinase; NeuN: neuronal nuclei; NF: neurofilament; SAPK: stress activated protein kinase; WB: Western blot analysis.

Quantification of the results was performed with the ImageJ 1.54 program (National Institutes of Health, Bethesda, MD, USA; Schneider et al., 2012). GADPH was used as a normalization control. To evaluate phosphoprotein levels, the ratio of the intensity of the phosphorylated protein band to that of the total protein band was determined and normalized to that of the control.

### Coimmunoprecipitation

Primary microglia were treated as described above. Microglia were lysed in 100 µL of lysate to each well and centrifuged for 15 minutes at 4°C and 12,000 × *g*. Next, A/G agarose beads (1:50, Thermo Fisher Scientific Inc.) and IgG antibody (1:500, Thermo Fisher Scientific Inc.) were added; the supernatant was collected, and A/G agarose beads (1:100) were added. CDNF primary antibody was added, and the samples were incubated for 12 hours at 4°C. The beads were rinsed with immunoprecipitation buffer and collected by centrifugation (5 minutes, 4°C, 3000 × *g*). The samples were boiled for 5 minutes and subjected to western blot analysis as described above.

### Animal model and intervention groups

Adult female BALB/c mice (4 weeks old, weight 15–18 g, *n* = 80) were acquired from the Experimental Animal Center of Shandong University. Mice were housed in a controlled environment with 12 hours light/dark cycles in a constant temperature of 23°C and humidity of 60%. Mice were randomly assigned to four groups: Sham group (*n* = 20), Lv-Vehicle (*n* = 20), Lv-CDNF (*n* = 20), and CDNF-shRNA (*n* = 20).

To establish the SCI model, anesthesia of mice was performed by inhalation of 3% isoflurane (Lunan Pharmaceuticals Co., Ltd., Linyi, China) and maintained with 1.5% isoflurane during the procedures. The complete removal of the lamina was done at the T10 level to reveal the thoracic spinal cord segment and undamaged dural sac. Spinal cord transection injury was performed with a #15 scalpel. The sham control group underwent T10 laminectomy without SCI.

After SCI, lentivirus (Lv-Vehicle, Lv-CDNF, and Lv-CDNF-shRNA, all from Genechem) were injected into two sites 5 mm proximal and distal from the SCI site (10 µL at each site) at five multiplicity of infection. The sham control group received an equivalent amount of phosphate-buffered saline. After closing the muscles and skin in layers, the mice were kept under a heat lamp to ensure their body temperature remained stable until they regained consciousness. Cefazolin (50 mg/kg, Qilu Pharmaceutical, Jinan, China) was given via intramuscular injection, and bladder reflex restoration was trained twice a day by manual bladder massage. All cell and animal experiments are illustrated in **Additional Figure 1**.

### Gait observation and behavioral assessments

The locomotor function of hindlimbs of mice was evaluated at 4 and 8 weeks after SCI. Hindlimb gait was observed. Footprints were recorded and assessed using the following parameters. (1) Bias of step length. Step length was measured as the distance from the heel of one footprint to the heel of the next footprint on the same side. The step-length bias was calculated as the ratio of the measured step length on the ipsilateral side to that on the contralateral side. (2) Footprint overlap bias. The distance between the center of the forefoot and hindfoot of the same step was measured. If the forefoot and hindfoot footprints overlapped, the footprint overlap was 0 cm. The footprint overlap bias was calculated by dividing the footprint overlap distance of the ipsilateral side by that of the contralateral side. (3) Hindfoot width. This parameter was measured as the distance from a point on the inner side of the hindfoot to the central axis. (4) Distance between hindfoot tracks. The distance between the hindfoot positions was measured between the midpoints of the two footprints for the same hindfoot step. Prior to the operation, the mice were introduced to the footprint track and initial measurements were taken (Zhou et al., 2020).

The open-field locomotor function was evaluated using the Basso Mouse Scale to account for the recovery pattern in the animals. The Basso Mouse Scale (score 0 to 9) is used to assess a wide variety of functions post-spinal impairments, ranging from partial joint movement, weight supported standing, coordinated walking, and trunk stability (Basso et al., 2006).

The horizontal ladder beam walking test was used to assess the hindlimb recovery in spinal contused mice, which was sensitive to determine the recovery of stepping and coordination. The ladder beam score was calculated by the ratio of positive events/total number of events. Positive events referred to successful steps while negative events included slips, missing steps, and dragging of steps (Cummings et al., 2007). All tests were performed by two independent investigators who were blinded to the treatments.

### Immunohistochemistry and immunofluorescence staining

Eight weeks after surgery, the animals were anesthetized by inhalation of 3% isoflurane and intracardially perfused with 4% paraformaldehyde. The entire spinal cord tissues were removed and morphologically observed. Tissues from the SCI site were embedded and sliced into 5-µm thick transverse sections. After staining with hematoxylin and eosin, the samples were subjected to immunohistochemistry with a primary antibody against anti-neurofilament (NF) and a secondary antibody conjugated with IRDye8. Photographs were taken with an upright microscope (Nikon Eclipse 80i, Tokyo, Japan). NF-positive nerve fibers were quantified using the ImageJ software.

Other samples were examined for the expression of CDNF and neuronal nuclei (NeuN). The samples were incubated with primary antibodies at 4°C overnight, followed by secondary antibodies conjugated with fluorescein isothiocyanate and Alexa 555 at 20°C for 2 hours. Cells were stained with 4′,6-diamidino-2-phenylindole (1:100, Cat#D1306, AB_2629482, Thermo Fisher Scientific) to visualize nuclei. Information of the primary and secondary antibodies is listed in **Additional Table 1**. Photographs were taken with a Nikon Eclipse 80i microscope and quantified using the ImageJ software.

### Enzyme-linked immunosorbent assay

Microglia were lysed and centrifuged to remove cell debris (15 minutes, 4°C, 12,000 × *g*). The Mouse TNF-α quantikine enzyme-linked immunosorbent assay (ELISA) kit, mouse IL-1β quantikine ELISA Kit, and mouse PGE2 quantikine ELISA kits from MultiSciences Biotech Co., Ltd. (Hangzhou, China) and CUSABIO (Wuhan, China) were used following the manufacturers’ guidelines. The optical density of each sample was measured at 450 nm using a Varioskan Flash Multimode Reader (ThermoFisher Scientific). The concentration of cytokines was determined using a standard curve.

### Real-time polymerase chain reaction

Total RNA was extracted from microglia using TRIzol reagent (Invitrogen, ThermoFisher Scientific). Two micrograms of RNA were converted into complementary DNA using a High-Capacity cDNA Reverse Transcription Kit (Applied Biosystems, ThermoFisher Scientific) following the manufacturer’s guidelines. Real-time polymerase chain reaction (RT-PCR) analysis was performed in triplicate using SYBR Green Master Mix (Applied Biosystems, ThermoFisher Scientific). The primers and RT-PCR conditions used for amplification have been described previously (Zhao et al., 2014). Confirmation of amplification specificity was achieved through melting curve analysis. Relative gene expression was calculated using the 2^–ΔΔCt^ method with the β-actin gene serving as an internal control (Zhang et al., 2019). The primers are listed in **Additional Table 2**.

**Additional Table 2:** The primers used in real-time polymerase chain reaction analysis

Primers	Forward sequence (5’-3’)	Reverse sequence (5’-3’)
IL-1β	CATGGAATCCGTGTCTTCCT	GAGCTGTCTGCTCATTCACG
TNF-α	CATCTTCTCAAAATTCGAGTGACAA	TGGGAGTAGACAAGGTACAACCC
PGES2	ACTGGCTGGTGCATCTCATC	TGCCAGGTCAGCAAGGTTAG

IL-1β: Interleukin-1 beta; PGES2: prostaglandin E synthase 2; TNF-α: tumor necrosis factor alpha.

### Statistical analysis

Statistical analysis was conducted using SPSS 20.0 (Version 20.0, IBM Corp., Armonk, NY, USA). Data were analyzed by one-way analysis of variance followed by Bonferroni’s *post hoc* test for multiple mean comparisons. Data are presented as mean ± standard deviation (SD). A *P*-value less than 0.05 indicated statistical significance.

## Results

### Molecular docking based on structural features of cerebral dopamine neurotrophic factor and JNK1

The three-dimensional structure and molecular docking calculations of CDNF and JNK1 were performed with AlphaFold3. The spatial structures of the proteins are shown in **Figure 1A**. The expected position error assessment (**Figure 1B**) showed reliability of the predicted 3D structure. Two domains in CDNF interact with JNK1 (**Figure 1C** and **D**). The C-terminal SAP domain of CDNF contacts JNK1 via five amino acids (LYS-148, GLN-149, GLN-152, GLU-157, and CYS-158), that interact with specific residues in JNK1 (GLN-120, VAL-219, and LYS-222) through hydrogen bonds. In the N-terminal saposin-like domain of CDNF, only one residue (LYS-94) interacts with JNK1 (at ASP-17) (**Figure 1E**).

### Cerebral dopamine neurotrophic factor combines to JNK1 and regulates JNK1/2 phosphorylation

**Figure 2:**
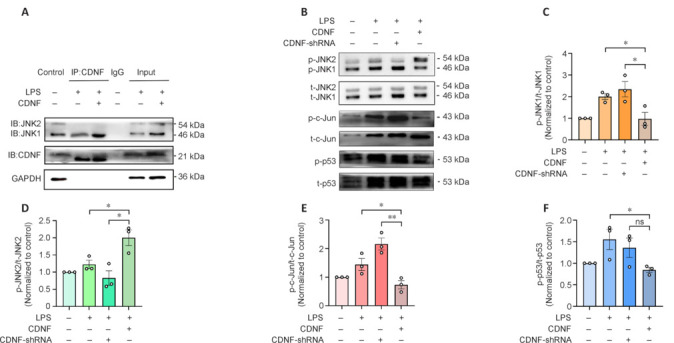
Interaction between CDNF and JNK1 in primary microglia. (A) Co-immunoprecipitation assay showed that JNK1 (46 kDa) was coprecipitated with CDNF. No binding between JNK2 (54 kDa) with CDNF was detected. (B) JNK1, JNK2, c-Jun, and p53 were evaluated by western blot. (C) Phosphorylation of JNK1 was induced by LPS stimulation and increased when CDNF was absent (CDNF-shRNA). CDNF significantly decreased the levels of p-JNK1 under LPS stress. (D) p-JNK2 increased in response to CDNF. (E) CDNF decreased c-Jun phosphorylation. (F) p53 phosphorylation was inhibited by CDNF. Data were obtained from experiments performed in triplicate and are expressed as mean ± SD. *P < 0.05, **P < 0.01 (one-way analysis of variance followed by Bonferroni’s *post hoc* test). CDNF: Cerebral dopamine neurotrophic factor; GAPDH: glyceraldehyde-3-phosphate dehydrogenase; JNK: c-Jun N-terminal kinase; LPS: lipopolysaccharide; shRNA: small hairpin RNA.

We next examined the interaction of CDNF and JNK1 in primary microglia established as described in Methods. Coimmunoprecipitation was used to determine the compound of CDNF and JNK1 or JNK2. The cells were treated with LPS to stimulate the inflammatory status, the exogenous CDNF was used to identify the combination between JNK and endogenous/exogenous CDNF. A blank control was treated without LPS or CDNF. The proteins drawn by A/G agarose beads with CDNF primary antibody then were performed immunoblot by JNK1 and JNK2 antibodies. The results showed that JNK1 (46 kDa) but not JNK2 (54 kDa) coprecipitated with CDNF. When the exogenous CDNF was added, the protein of JNK1 was correspondingly increased, indicating that JNK1 could combine to both endogenous and exogenous CDNF (**Figure 2A**). Next, we examined the phosphorylation of JNK1 and JNK2 under the regulation of CDNF using western blot analysis (**Figure 2B–D**). The results showed that the activation of p-JNK1 was induced by LPS treatment, and this phosphorylation was enhanced in cells treated with CDNF-shRNA. CDNF administration decreased the level of p-JNK1 under LPS (CDNF + LPS *vs.* LPS, *P* = 0.0418, LPS + CDNF-shRNA *vs*. LPS + CDNF, *P* = 0.0358; **Figure 2C**). Notably, CDNF demonstrated its role in increasing p-JNK2 levels (CDNF + LPS *vs.* LPS, *P* = 0.0391; LPS + CDNF-shRNA *vs*. LPS + CDNF, *P* = 0.0191; **Figure 2D**). The phosphorylation of c-Jun was inhibited by CDNF treatment (CDNF + LPS *vs.* LPS, *P* = 0.0150, LPS + CDNF-shRNA *vs.* LPS + CDNF, *P* = 0.0051; **Figure 2E**), and the phosphorylation of p53 was also alleviated by CDNF intervention (CDNF + LPS *vs.* LPS, *P* = 0.0468; **Figure 2F**). These results indicate that CDNF regulates JNK1/2 by inhibiting p-JNK1 and increasing p-JNK2 levels.

### Cerebral dopamine neurotrophic factor improves behavioral recovery of spinal cord injury model mice

**Figure 3:**
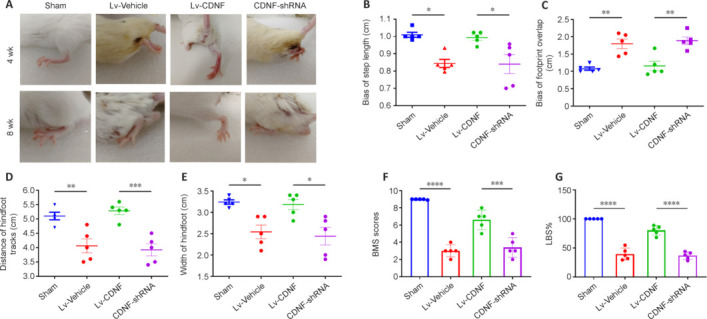
CDNF improves behavioral functional recovery of SCI model mice. (A) At 4 weeks after injury, comparing to the Sham group, the hindlimbs of mice in Lv-Vehicle group showed inability to support weight. While the hindlimbs of the Lv-CDNF group showed partial ability to support weight comparing to CDNF-shRNA group. At 8 weeks after injury, comparing to the Sham group, the Lv-Vehicle group showed partial recovery to support weight. In the Lv-CDNF group, the recovery of locomotor function and weight support of hindlimbs was obviously superior to CDNF-shRNA group. (B–E) Footprint parameters were measured and assessed to evaluate gait recovery. (B) At 8 weeks post-injury, the bias of step length in Lv-Vehicle group was significantly lower than Sham, and Lv-CDNF showed higher scores *versus* CDNF-shRNA group. (C) The bias of footprint overlap was significantly greater in Lv-Vehicle group compared with Sham group, but Lv-CDNF reduced the scores of CDNF-shRNA group. The distance between the hindfoot prints (D) and the width of the hindfoot (E) were consistent with the bias of the step length. (F) The BMS scores were reduced apparently after spinal cord injury with Lv-Vehicle. But higher BMS scores were observed in Lv-CDNF group than CDNF-shRNA group. (G) The LBS% decreased after SCI in models, however, higher LBS% was observed in the Lv-CDNF group compared with the CDNF-shRNA group. Data are expressed as mean ± SD (*n* = 5). *P < 0.05, **P < 0.01, ***P < 0.001 (one-way analysis of variance followed by Bonferroni’s *post hoc* test). BMS: Basso Mouse Scale; CDNF: cerebral dopamine neurotrophic factor; LBS%: ladder beam score; Lv: lentivirus; SCI: spinal cord injury; shRNA: small hairpin RNA.

We next examined the effects of CDNF on SCI model mice using lentivirus overexpressing CDNF or lentivirus expressing shRNA targeting CDNF. The sham control group exhibited normal locomotor and sensorimotor functions. Following SCI, mice in all groups exhibited an immediate loss of hind limb function, displaying a pattern of moving forward with the forelimbs and placing the hind limbs without bearing weight (**Figure 3A**). Four weeks after the injury, the hind limbs of mice in the Lv-Vehicle group still exhibited an inability to support their weight, compared to the normal performance observed in the Sham group. In the Lv-CDNF group, the hind limbs showed locomotor recovery and partial ability to support body weight, while the CDNF-shRNA group showed the inability of hind limbs. Eight weeks after the injury, the hind limbs of the CDNF-shRNA and Lv-Vehicle groups had some ability to support weight, compared to the Sham group. The Lv-CDNF group showed a restoration of hind limb locomotor function and weight-bearing ability.

Footprint parameters were measured and assessed to evaluate gait recovery. Eight weeks post-injury, the step-length bias in the Lv-Vehicle group demonstrated significantly lower levels compared with the Sham group (*P* = 0.0120). Compared with the low levels observed in the CDNF-shRNA group, the Lv-CDNF group demonstrated a greater step-length bias, indicating improved walking function (*P* = 0.0218; **Figure 3B**). The footprint overlap bias in the Lv-CDNF group was significantly lower than that in the CDNF-shRNA group (*P* = 0.0018). In contrast, the Lv-Vehicle group showed higher scores compared with the Sham group (*P* = 0.0021; **Figure 3C**). The distance of the hindfoot tracks was significantly greater in the Lv-CDNF group compared with the CDNF-shRNA group (*P* = 0.0005). Conversely, the Lv-Vehicle group exhibited lower scores compared to the Sham group (*P* = 0.0066; **Figure 3D**). Consistently, the width of the hindfoot prints in the Lv-CDNF group was greater than in the CDNF-shRNA group (*P* = 0.0148), while the Lv-Vehicle group showed a lower width compared with the Sham group (*P* = 0.0224; **Figure 3E**).

Open-field locomotor assessments using the Basso Mouse Scale showed a higher score in the Lv-CDNF group compared with the CDNF-shRNA group (*P* = 0.0002), and Lv-Vehicle group showed lower scores compared with the Sham group (*P* = 0.0001; **Figure 3F**). The ladder beam score reflects the ratio of positive events to total events during a ladder beam walking task. The results demonstrated a higher ladder beam score in the Lv-CDNF group compared with the CDNF-shRNA group (*P* = 0.0001), while the Lv-Vehicle group had a lower score compared with the Sham group (*P* = 0.0001; **Figure 3G**). These findings indicate that CDNF exerted a positive impact on aiding behavioral recovery post-SCI in mice.

### Cerebral dopamine neurotrophic factor alleviates spinal cord pathological injury in spinal cord injury model mice

**Figure 4:**
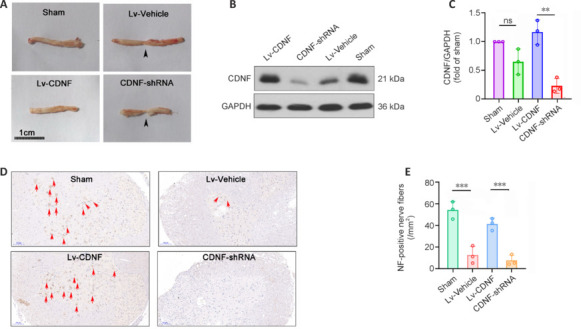
CDNF alleviates spinal cord pathological injury in SCI model mice. (A) Spinal cord tissues were harvested from the injury site at 8 weeks and subjected to determination of immunoblotting and immunohistochemistry observation. Damaged sites are indicated by arrows. (B) Western blot of the tissues from the spinal cord injury sites confirmed the overexpression of CDNF in the Lv-CDNF group and the scramble of CDNF in CDNF-shRNA group. The CDNF levels in the Lv-Vehicle group showed no statistical difference compared with the Sham group. (C) Quantitative analysis of CDNF in spinal cord tissue of the four groups. (D) Regenerated nerve fibers were detected by NF antibody; positive staining is shown in brown (red arrows). Scale bars: 100 µm. (E) Quantification of NF staining in the indicated groups. The Lv-CDNF group showed more NF-positive neural fibers than the Lv-Vehicle group. Data are expressed as mean ± SD (*n* = 3). *P < 0.05, **P < 0.01, ***P < 0.001 (one-way analysis of variance followed by Bonferroni’s *post hoc* test). CDNF: Cerebral dopamine neurotrophic factor; Lv: lentivirus; NF: neurofilament; SCI: spinal cord injury; shRNA: small hairpin RNA.

To examine the restoration and rearchitecture of the damaged tissue of the spinal cord after injury, histological and immunohistochemical assays were performed. Spinal cord tissues were harvested from the injury site at 8 weeks post modeling revealed (**Figure 4A**). Compared to the Sham group, the site of spinal cord injury was observed in both the Lv-Vehicle and CDNF-shRNA groups, while recovery of the damaged site was noted in the Lv-CDNF group. Western blot of the tissue harvested from the SCI site confirmed efficient lentivirus-mediated expression of CDNF or CDNF scrambled by shRNA (**Figure 4B**). CDNF expression was knocked down by CDNF-shRNA (**Figure 4C**).

To identify newly formed nerve fibers in the spinal cord, immunohistochemistry for NF was conducted (**Figure 4D**). Compared with the Sham group, the Lv-Vehicle group exhibited poor nerve fiber regeneration. In contrast, the Lv-CDNF group showed a significantly higher level of neural fibers compared to the CDNF-shRNA group, indicating an improvement in neural regeneration (**Figure 4E**).

### Cerebral dopamine neurotrophic factor improves the recovery of the injured spinal cord in spinal cord injury model mice

**Figure 5:**
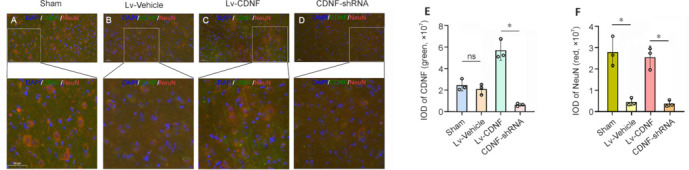
CDNF improves the recovery of the injured spinal cord in SCI model mice. (A–D) The rearchitecture of neurons at the site of SCI was assessed by NeuN immunofluorescence. NeuN (red), CDNF (green), and DAPI (blue). Scale bars: 50 µm. (E) The overexpression and downregulation of CDNF was confirmed in Lv-CDNF and CDNF-shRNA groups, respectively. (F) NeuN staining was significantly higher in the Lv-CDNF group than the Lv-Vehicle group. Data are expressed as mean ± SD (*n* = 3). *P < 0.05 (one-way analysis of variance followed by Bonferroni’s *post hoc* test). CDNF: Cerebral dopamine neurotrophic factor; DAPI: 4′,6-diamidino-2-phenylindole; IOD: integrated optical density; Lv: lentivirus; NeuN: neuronal nuclei; SCI: spinal cord injury; shRNA: small hairpin RNA.

The rearchitecture of neurons at the site of SCI was assessed by NeuN immunofluorescence (**Figure 5A–D**). NeuN expression was lower in the Lv-Vehicle group compared with the sham group, indicating neuron damage post-SCI. The overexpression and scramble of CDNF in the Lv-CDNF and CDNF-shRNA groups were confirmed (**Figure 5E**). The CDNF levels in the Lv-Vehicle group showed no statistical difference compared with the Sham group. The higher expression of NeuN was observed in the Lv-CDNF group compared with CDNF-shRNA group (**Figure 5F**), demonstrating that Lv-CDNF promoted the recovery of neurons in the injured spinal cord.

### Cerebral dopamine neurotrophic factor decreases the transcription and secretion of proinflammatory cytokines in lipopolysaccharide-treated microglia

**Figure 6:**
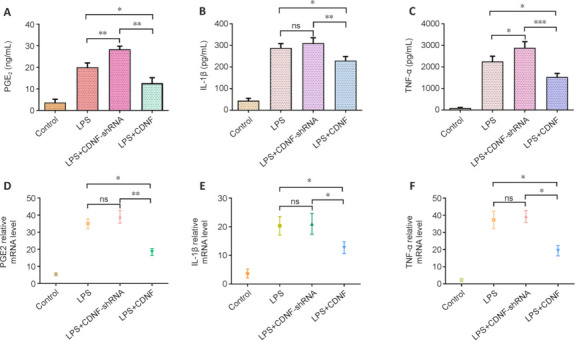
CDNF decreases the transcription and secretion of proinflammatory cytokines in LPS-treated microglia. (A–C) The release of proinflammatory mediators PGE2, IL-1β and TNF-α was provoked by LPS. Knockdown of the CDNF further enhanced the secretion of PGE2 and TNF-α. CDNF administration significantly decreased the stimulation of these cytokines. (D–F) Real-time PCR assays revealed a notable rise in PGE2, IL-1β, and TNF-α mRNA transcription in microglia under LPS exposure. CDNF treatment reduced the mRNA levels of PGES2, IL-1β and TNF-α in LPS-induced microglia. Data are expressed as mean ± SD (*n* = 3). *P < 0.05, **P < 0.01, ***P < 0.001 (one-way analysis of variance followed by Bonferroni’s *post hoc* test). CDNF: Cerebral dopamine neurotrophic factor; IL-1β: interleukin-1 beta; LPS: lipopolysaccharide; ns: not significant; PGE2: prostaglandin E2; PCR: polymerase chain reaction; SCI: spinal cord injury; shRNA: small hairpin RNA; TNF-α: tumor necrosis factor.

To examine the influence of CDNF on neuroinflammation in microglia, the levels of the inflammatory cytokines PGE2, IL-1β, and TNF-α in the supernatant of cultured microglia exposed to LPS were measured using ELISA. LPS greatly stimulated the release of PGE2, TNF-α, and IL-1β (**Figure 6A–C**). Notably, knockdown of CDNF increased the neuroinflammation induced by LPS. Conversely, CDNF significantly decreased the levels of the three proinflammatory cytokines. RT-PCR showed significantly increased mRNA levels of the proinflammatory cytokines PGE2, IL-1β, and TNF-α following LPS stimulation (**Figure 6D–F**). CDNF-shRNA significantly increased the mRNA levels of the three cytokines in microglia treated with LPS, while CDNF reduced the mRNA levels.

### The signal transduction pathways of cerebral dopamine neurotrophic factor in microglia by targeting JNK1 and regulating the balance of JNK1/JNK2

**Figure 7:**
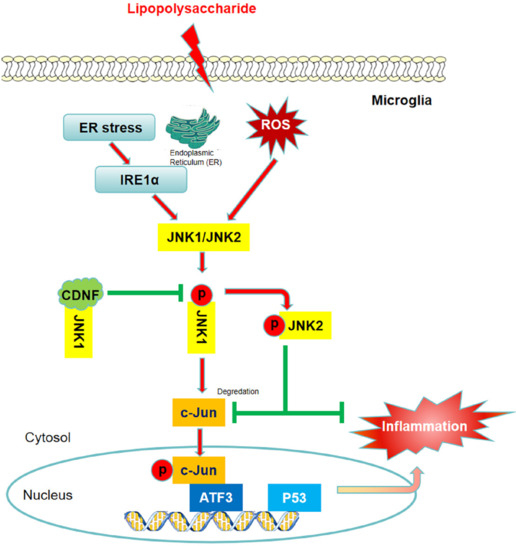
CDNF alleviates inflammation in microglia by targeting JNK1 and regulating the balance of JNK1/JNK2. The hypothesis of signal transduction pathways is supplemented by the interaction between CDNF and JNK1. This binding may competitively inhibit the phosphorylation of JNK1 following ER stress or ROS under injury status. The activation of c-Jun is induced by p-JNK1, leading to the initiation of apoptosis and proinflammatory factor transcription. As a result of the repressed activity of JNK1, the following inflammatory responses were inhibited. Meanwhile JNK2 becomes more active to exert neuroprotective effects. In summary, CDNF suppresses the activation of c-Jun and p53 by targeting JNK1 and regulating JNK2, resulting in the restrain of the production of inflammatory cytokines during neuroinflammation. ATF3: Activating transcription factor 3; CDNF: cerebral dopamine neurotrophic factor; ER: endoplasmic reticulum; JNK: c-Jun N-terminal kinase; ROS: reactive oxygen species.

Together, these findings support the hypothesis that CDNF targets JNK1 within signal transduction pathways (**Figure 7**). This binding may competitively inhibit the phosphorylation of JNK1 in response to ER stress or ROS under injury conditions. The activation of c-Jun is induced by p-JNK1, leading to the initiation of apoptosis and the transcription of pro-inflammatory factors. As a result of the repressed activity of JNK1, subsequent inflammatory responses are inhibited. Meanwhile, JNK2 becomes more active, exerting neuroprotective effects. In summary, CDNF suppresses the activation of c-Jun and p53 by targeting JNK1 and regulating JNK2, thereby restraining the production of inflammatory cytokines during neuroinflammation.

## Discussion

Neuroinflammation is a key driver of pathology in traumatic SCI and various neurodegenerative diseases, including Alzheimer’s disease (Leng and Edison, 2021; Li et al., 2022; Teleanu et al., 2022). Persistent activation of glial cells, particularly microglia and astrocytes, has been documented in neurodegenerative models, where these cells produce proinflammatory cytokines and neurotoxic mediators that contribute to the progression of neural damage (Gao et al., 2023). In SCI, pathological factors such as ER stress, blood-spinal cord barrier disruption, ischemia-reperfusion injury, and ROS accumulation exacerbate this inflammatory cascade, creating a hostile microenvironment that impedes neural repair (Fan et al., 2015; Yu et al., 2016; Li et al., 2019a; Jin et al., 2021; Whittemore et al., 2022; Yin et al., 2024). Our findings, demonstrating increased microglial activation in a SCI mouse model, corroborate prior observations linking chronic neuroinflammation to sustained tissue damage and impaired recovery. The dual role of glial cells in maintaining central nervous system homeostasis and mediating injury responses highlights the complexity of neuroinflammation. While glial activation is essential for clearing debris and promoting initial repair, uncontrolled or sustained activation can lead to a self-perpetuating inflammatory cycle. Proinflammatory cytokines such as TNF-α, IL-1β, and IFN-α, along with neurotoxic molecules like ROS and PGE2, contribute to secondary injury, further disrupting the microenvironment. PGE2, in particular, has been implicated in amplifying inflammatory reactions through its role as an arachidonic acid derivative. The persistent presence of these factors following SCI underscores the need for interventions targeting both inflammation and the adverse microenvironment to facilitate neural regeneration (Orr and Gensel, 2018; Clifford et al., 2023).

Axonal regeneration and functional recovery of the spinal cord can only be promoted if the microenvironment of the traumatic lesion is improved. Glial scarring, primarily driven by activated astrocytes, represents a major barrier to axonal regeneration and functional recovery in SCI. The formation of these scars post inflammation not only creates a physical obstruction but also reinforces molecular barriers that inhibit axon growth. Consistent with prior studies, we observed that the microenvironment surrounding SCI lesions deteriorates as a result of the sustained release of inflammatory mediators and local neuroinflammation. Importantly, our findings reveal that administration of CDNF mitigates the inflammation after spinal cord injury, preventing the scar formation, and promoting a more favorable environment for regeneration (**Additional file 1**). The mechanism of action of CDNF provides critical insights into its potential as a therapeutic agent. As an ER-localized protein, CDNF alleviates ER stress, a well-documented contributor to neuroinflammation and neuronal apoptosis (Lindholm et al., 2007; Eremin et al., 2021; Lõhelaid et al., 2024). The therapeutic potential of CDNF has been testified and the neuroprotective and neurorestorative effects have been described in various disease models, e.g., Parkinson’s disease, stroke and amyotrophic lateral sclerosis (Lõhelaid et al., 2024).

The suppression of JNK1 activity by CDNF and the subsequent inhibition of c-Jun phosphorylation provide a plausible mechanism for the anti-inflammatory and neuroprotective effects of CDNF. As a key component of the activator protein-1 transcription factor complex, c-Jun regulates the expression of pro-apoptotic and proinflammatory mediators, including p53, Bcl-2 family members and cyclooxygenase-2 (Zhang and Zhang, 2005; Ibrahim et al., 2022). By inhibiting c-Jun activation, CDNF may attenuate these harmful pathways, reducing neuronal loss and dampening neuroinflammation. Notably, this mechanism parallels findings with MANF, a related neurotrophic factor, whose C-terminal SAP domain has been shown to independently promote neuronal survival (Hellman et al., 2011). The structural similarity between CDNF and MANF underscores the importance of the SAP domain in neurotrophic factor-mediated neuroprotection.

The present study also expands on the neuroprotective potential of CDNF by elucidating its interaction with JNK1 and JNK2 in the context of SCI. While previous studies have focused on CDNF’s anti-inflammatory and neuroprotective properties in other disease models, this study demonstrates the ability of CDNF to modulate specific signaling pathways critical to SCI pathology. By addressing both the inflammatory response and the adverse microenvironment, CDNF offers a comprehensive approach to neural repair. The CDNF protein consists of two domains: an N-terminal saposin-like domain and a C-terminal SAF-A/B, Acinus, and PIAS (SAP) domain (Lindholm and Saarma, 2022). In this study, we demonstrated that CDNF interacts with JNK1 primarily through its C-terminal SAP domain, suppressing JNK1 phosphorylation while inducing JNK2 activation. Our findings indicate that five amino acids in the C-terminal SAP domain of CDNF form hydrogen bonds with JNK1, while only one amino acid in the N-terminal saposin-like domain contributes to this interaction. This is consistent with previous research showing that the C-terminal SAP domain exhibits independent neuroprotective activity, similar to mesencephalic astrocyte-derived neurotrophic factor (MANF), by promoting neuronal survival *in vitro* (Parkash et al., 2009). Notably, the neuroprotective activity of MANF was lost when the CXXC motif in the C-terminal SAP domain was mutated (Mätlik et al., 2015).

JNKs are members of the mitogen-activated protein kinase (MAPK) family. JNK1 and JNK2 display opposite functions, with JNK1 stimulating apoptosis and JNK2 activity favoring cell survival (Lu et al., 2023). In multiple myeloma cell lines, JNK2 plays critical roles in the maintenance of cell survival and suppresses JNK1-mediated apoptosis (Barbarulo et al., 2013). In cancer cells, the apoptosis induced by proteasome inhibitor requires JNK1 but is inhibited by JNK2 (Pietkiewicz et al., 2013). Our data on modulation of JNK isoforms by CDNF is consistent with reports of their opposing roles in cellular stress responses. While JNK1 activity drives apoptosis under stress conditions, JNK2 has been shown to support cell survival and counteract JNK1-mediated apoptosis.

JNK1 deficiency results in reduced phosphorylation and stability of c-Jun, while JNK2 deficiency leads to increased phosphorylation and stability of c-Jun. Re-expression of JNK2 in JNK2^–/–^ cells reversed the null phenotype, whereas ectopic expression of JNK1 enhanced it. JNK2 preferentially binds to c-Jun in unstimulated cells, promoting c-Jun degradation. In contrast, JNK1 becomes the primary c-Jun-interacting kinase upon cell stimulation. These data provide mechanistic insights into the differential effects of the various JNK isoforms (Sabapathy et al., 2004). By modulating these pathways, CDNF shifts the balance toward neuroprotection and repair. This study contributes to our understanding by demonstrating that CDNF regulates the activation of JNK1 and JNK2, further tipping the balance toward survival pathways. These findings suggest that the dual modulation of JNK isoforms of CDNF represents a novel therapeutic approach for mitigating ER stress-induced damage and promoting neuronal recovery.

The microenvironment of the injured spinal cord is profoundly disrupted at the cellular and molecular levels after SCI. This imbalance manifests as glial activation, oligodendrocyte loss, and axonal demyelination, all of which impair neuronal regeneration. Our results indicate that CDNF ameliorates these events by reducing glial activation and promoting remyelination. NeuN and NF immunohistochemical analyses showed enhanced neuronal survival and axonal regeneration in CDNF-treated groups, consistent with reports that reducing inflammation can restore neural function (DiSabato et al., 2016). Following SCI, the equilibrium in the microenvironment of the spinal cord is disturbed, resulting in a cascade of physiological alterations (Ahuja et al., 2017; Fan et al., 2018). The imbalance in this small-scale environment happens at three different stages and degrees – molecules, cells, and tissues – at various points in time. At the cellular level, this imbalance includes the activation of astrocytes, the differentiation of endogenous neural stem cells, oligodendrocyte progenitors, and microglia, and the infiltration of macrophages. At the tissue level, these changes play a role in bleeding, lack of blood supply, the creation of glial scars, loss of myelin, and the restoration of myelin. At the molecular level, these changes play a role in the production of neurotrophic factors and their precursor peptides, cytokines, and chemokines. Eventually the disruption of the microenvironment impairs nerve regeneration and functional recovery (Fan et al., 2018).

Glial scars result from astrocyte proliferation in the injured areas (Pekny and Nilsson, 2005). The connection between large cell bodies and astrocytic protrusions creates a barrier, both physically and molecularly, that obstructs the regeneration and growth of axons (Sofroniew, 2009). The scar continues to strengthen as cysts and cavities develop over a period of six months following the injury (Rowland et al., 2008; Siddiqui et al., 2015). Following SCI, axon demyelination occurs as a result of direct injury and an imbalance in the surrounding microenvironment, primarily caused by the death of oligodendrocytes through necrosis and apoptosis. Demyelination is caused by inflammation and immune dysfunction in the central nervous system, which often leads to functional disability (Li et al., 2019b; Klotz et al., 2023). Oligodendrocyte death is caused by traumatic injury, lack of blood flow, inflammation, oxidative damage, and cellular self-destruction as a result of an imbalance in the surrounding environment (Lee et al., 2015; Jacquens et al., 2022; Zirngibl et al., 2022). The microenvironment may be improved by regulating inflammation at the SCI site where CDNF is expressed, allowing it to play an anti-inflammatory role. Inhibiting the activation of glial cells, such as astrocytes and microglia, limits the development of glial scars.

NeuN is localized in the nucleus and perinuclear cytoplasm of neurons in the mammalian central nervous system. Immunohistochemical analysis of NeuN has been used to evaluate the functional status of neurons in both normal and pathological situations. Notably, in some studies, the decrease in NeuN immunoreactivity was attributed to neuronal demise (Duan et al., 2016). In a previous report, NeuN immunostaining and terminal deoxynucleotidyl transferase dUTP nick-end labeling (TUNEL) staining were performed in an ischemic setting, revealing a significant decrease in NeuN immunoreactivity 24 hours post-exposure that was associated with a rise in apoptotic cell count (Unal-Cevik et al., 2004). These data indicate that the reduction in NeuN staining is linked to the death of neurons in affected regions of the central nervous system. In the current study, NeuN expression analysis showed that CDNF promoted the recovery of neurons, and the regenerated nerve fibers were positive for NF immunohistochemistry in the spinal cord.

This study has several limitations. First, while this study elucidated the interactions of CDNF with JNK1 and JNK2, it primarily relied on *in vitro* and structural modeling data to propose these mechanisms. Additional *in vivo* validation using techniques such as genetic knockout mouse models would provide more definitive evidence of CDNF’s role in neuroinflammation regulation. Second, further research is needed to fully understand the molecular mechanisms underlying the effects of CDNF, particularly its interaction with other signaling pathways involved in SCI recovery. Translational studies will also be essential to validate the efficacy and safety of CDNF in clinical settings.

In summary, the current study reveals that CDNF interacts with JNK1 through the C-terminal SAP domain and regulates the JNK1/2-c-Jun-p53 pathway (**Figure 7**). Our data reveal a neuroprotective role of CDNF through its improving the microenvironment via its anti-inflammatory effects. CDNF expression reduces the release of inflammatory cytokines and alleviates neuroinflammation, potentially creating a favorable environment for histological and functional recovery for SCI. This study provides evidence for CDNF, regulating the neuroinfalmmation by targeting JNK1 in spinal cord injury.

## Additional files:

***Additional Figure 1:*** *Flowcharts for the cell (A) and animal (B) experiments.*

Additional Figure 1 Flowcharts of the cell (A) and animal (B) experiments
CDNF: Cerebral dopamine neurotrophic factor; Co-IP: coimmunoprecipitation; ELISA: enzyme-linked immunosorbent assay; JNK: c-Jun N-terminal kinase; LPS: lipopolysaccharide; Lv: lentivirus; RT-PCR: real-time polymerase chain reaction; shRNA: small hairpin RNA.

***Additional Table 1:*** *The antibodies used in this study.*

***Additional Table 2:*** *The primers used in real-time polymerase chain reaction analysis.*

***Additional file 1:*** *The regulation of microglial activation and neuroinflammation post spinal cord injury.*

Additional file 1 The regulation of microglial activation and neuroinflammation post spinal cord injury

## Data Availability

*The RNA sequencing data of this study are openly available in GEO at https://www.ncbi.nlm.nih.gov/geo/. The data generated or analyzed during this study are included in this published article and the Additional files*.
