# Supplementary figures and images for: Cerebral dopamine neurotrophic factor for spinal cord injury: Targeting JNK1 to relieve neuroinflammation and improve neural repair
Source: Neural Regen Res. 2025 Jun 19;21(7):3114–21. doi: 10.4103/NRR.NRR-D-24-00890 (PMC13378946; doi:10.4103/NRR.NRR-D-24-00890)

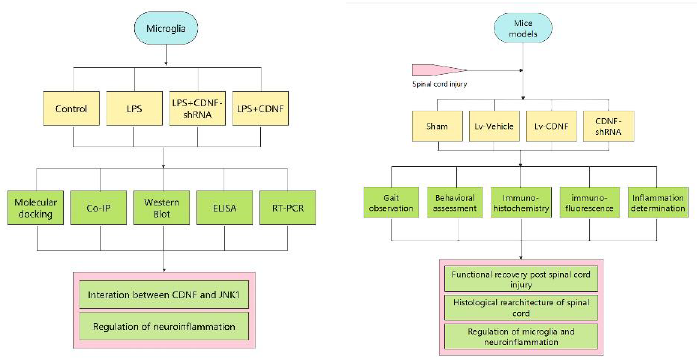

Supplement: Supplementary file 1 [file NRR-21-3114_Suppl1.tif]
