# Supplementary material for: Cerebral dopamine neurotrophic factor for spinal cord injury: Targeting JNK1 to relieve neuroinflammation and improve neural repair
Source: Neural Regen Res. 2025 Jun 19;21(7):3114–21. doi: 10.4103/NRR.NRR-D-24-00890 (PMC13378946; doi:10.4103/NRR.NRR-D-24-00890)
Supplement: Supplementary file 2 [file NRR-21-3114_Suppl1.pdf]

Additional file 1 The regulation of microglial activation and neuroinflammation post spinal cord injury

Materials and methods

Determination of microglia activation and inflammation in spinal cord

The samples of spinal cord were harvested and sliced into frozen sections at 8 weeks after modeling. The ionized calcium-binding adapter molecule 1 (Iba1) antibody conjugated with Alexa Fluor 594 and the antibodies of CDNF were utilized in the experiments. All antibodies were listed in Additional Table 1. ImageJ was used for measuring the integrated optical density of Iba1-positive areas, which acted as the marker of microglial activation. The same amounts of spinal cord tissues were taken from the injured site and the homogenate was ground in the extraction buffer in the ice bath. Homogenate was centrifuged at 5,000 × *g*, at 4°C, for 15 minutes. Remove supernatant and store at -80°C. The levels of IL-1β were determined by ELISA kit (MultiSciences Biotech Co., Ltd., Hangzhou, China).

Results

Microglia activation and proinflammatory cytokines expression in spinal cord injury

Immunostaining results showed that more microglia were activated in all spinal cord injury groups than in Sham group, among which Lv-CDNF group could reduce the activation degree of microglia and alleviate the inflammatory response to a certain extent, comparing to CDNF-shRNA and Lv-Vehicle groups (**Figure 1A-E**). The ELISA result showed that SCI modeling provoked the IL-1β levels. Lv-CDNF transduction could alleviate this proinflammatory secretion, however, the comparison between Lv-CDNF and CDNF-shRNA, Lv-Vehicle groups worked out with no significant difference statistically (**Figure 1F**). This might due to the sampling time at 8 weeks after the modeling, the secretion of inflammatory factors had been stabilized, so that levels of inflammatory factors such as IL-1 in the tissues might not be dramatically different.

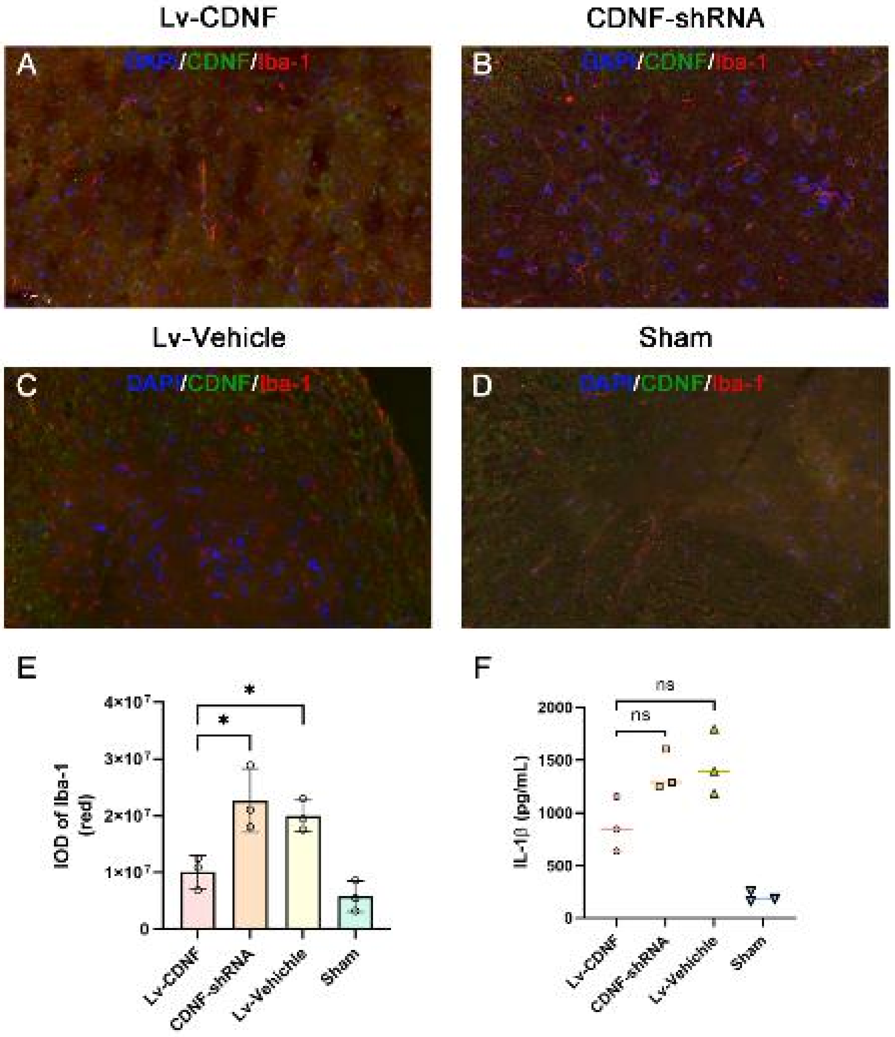

Figure 1. Microglia activation and IL-1β increase in spinal cord.

(A-D) Iba1 (red, Alexa Fluor 594) was detected as a marker of activated microglia, along with CDNF (green, FITC) and DAPI. The expression of Iba1 was significantly higher in spinal cord injury groups comparing to sham group and Lv-CDNF alleviated this upregulation. Scale bar: 50 µm. (E) Statistical graph showed that IOD of Iba1 was higher in the spinal cord injury model groups, indicating that microglia was activated and this activation could be reduced by Lv-CDNF. (F) IL-1β levels determined by ELISA showed that SCI modeling provoked the IL-1β levels. Lv-CDNF transduction could alleviate this proinflammatory secretion, however, the difference was not significant statistically. Data are expressed as mean ± SD (n = 3). *P < 0.05 (one-way analysis of variance followed by Bonferroni test). CDNF: Cerebral dopamine neurotrophic factor; DAPI: 4’,6-diamidino-2-phenylindole; ELISA: enzyme-linked immunosorbent assay; FITC: fluorescein isothiocyanate; Iba1: ionized calcium-binding adapter molecule 1; IOD: integrated optical density; Lv: lentivirus; ns: not significant; shRNA: small hairpin RNA.
